# Chemokines and their receptors in Atherosclerosis

**DOI:** 10.1007/s00109-015-1317-8

**Published:** 2015-07-15

**Authors:** Emiel P. C. van der Vorst, Yvonne Döring, Christian Weber

**Affiliations:** Institute for Cardiovascular Prevention, Ludwig-Maximilians-University Munich, Pettenkoferstr 9, 80336 Munich, Germany; DZHK (German Centre for Cardiovascular Research), partner site Munich Heart Alliance, Munich, Germany; Cardiovascular Research Institute Maastricht (CARIM), Maastricht University, Maastricht, The Netherlands

**Keywords:** Cardiovascular disease, Atherosclerosis, Chemokines, Macrophage migration inhibitory factor, CXCL12

## Abstract

Atherosclerosis, a chronic inflammatory disease of the medium- and large-sized arteries, is the main underlying cause of cardiovascular diseases (CVDs) most often leading to a myocardial infarction or stroke. However, atherosclerosis can also develop without this clinical manifestation. The pathophysiology of atherosclerosis is very complex and consists of many cells and molecules interacting with each other. Over the last years, chemokines (small 8–12 kDa cytokines with chemotactic properties) have been identified as key players in atherogenesis. However, this remains a very active and dynamic field of research. Here, we will give an overview of the current knowledge about the involvement of chemokines in all phases of atherosclerotic lesion development. Furthermore, we will focus on two chemokines that recently have been associated with atherogenesis, CXCL12, and macrophage migration inhibitory factor (MIF). Both chemokines play a crucial role in leukocyte recruitment and arrest, a critical step in atherosclerosis development. MIF has shown to be a more pro-inflammatory and thus pro-atherogenic chemokine, instead CXCL12 seems to have a more protective function. However, results about this protective role are still quite debatable. Future research will further elucidate the precise role of these chemokines in atherosclerosis and determine the potential of chemokine-based therapies.

## Introduction

Atherosclerosis is the main underlying cause of cardiovascular disease (CVD) [1], the leading cause of death worldwide accounting for more than 15 million deaths annually [2]. Most commonly, CVD results in a myocardial infarction (MI) or stroke but can also develop without clinical manifestations. Over the years, a lot of research has been performed to better understand the pathology behind CVDs. Atherosclerosis is a chronic inflammatory disease, characterized by the accumulation of lipids, immune cells, and cell debris in the vessel wall. This will form atherosclerotic lesions that can grow over time and eventually occlude an artery. However, more frequently, these lesions rupture, causing thrombus formation [1]. The occlusion will cause ischemia in downstream tissues, resulting in cardiovascular events. At present, atherosclerosis cannot be reversed by medical treatment, warranting the need for better understanding of this pathology in order to develop new strategies to combat this deathly disease. In this review, we will specifically focus on the role of chemokines and their receptors in atherosclerosis, as they have been shown to play crucial roles in the initiation, progression, and even regression of atherosclerotic lesions. Finally, CXCL12 and macrophage migration inhibitory factor (MIF), which have been recently associated with CVD, will be more elaborately discussed.

## Chemokines

**Fig. 1:**
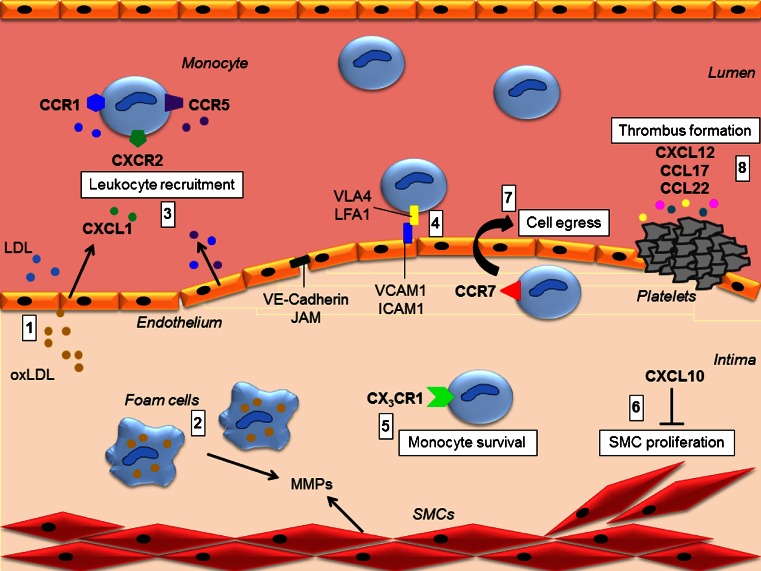
Role of chemokines in atherosclerosis. After endothelial cell damage, LDL will migrate into the intima, where it will undergo oxidation (*1*). Modified LDL will subsequently be taken up by macrophages, forming foam cells (*2*). Lysophosphatidic acid, a component of LDL, induces endothelial CXCL1 secretion to recruit monocytes via CXCR2. CCR1 and CCR5 are crucial chemokine receptors for leukocyte recruitment (*3*). Recruited leukocytes will adhere to the vessel wall, using VCAM-1-VLA4 and ICAM1-LFA1 interactions (*4*). Continuous accumulation of lipids and debris will eventually result in apoptosis of lesional macrophages. CX_3_CR1 is important for monocyte survival, by protecting them from apoptosis (*5*). Upon lesion progression, a fibrous cap is formed to protect the lumen from the necrotic core. This cap consists of collagen and elastin, produced by SMCs. CXCL10 inhibits SMC proliferation, thereby influencing plaque stability (*6*). CCR7 can mediate monocyte/macrophage egress from lesions (*7*). Upon lesion rupture, platelets adhere to the site of injury, and a thrombus is formed (*8*). CXCL12, CCL17, and CCL22 play an important role in platelet activation and aggrevation. *ICAM1* intercellular adhesion molecule 1, *JAM* junctional adhesion molecule, *LDL* low-density lipoproteins, *LFA1* lymphocyte function-associated antigen 1, *MMP* matrix metalloproteases, *SMCs* smooth muscle cells, *VCAM1* vascular cell adhesion molecule 1, *VLA4* very late antigen 4

Chemokines are small molecules (8–12 kDa) that are the largest family of cytokines [3]. Based on the position of the N-terminal cysteine residues, this family can be divided into four canonical subclasses, being C, CC, CXC, and CX_3_C [4]. Characteristic for all chemokines is that they exert chemotactic effects on cells, in contrast to regular cytokines like IL-10 or IL-12 that do not mediate this cell attraction. Recently, a group of cytokines was identified that share functional similarities with chemokines as they exert some chemokine-like functions like chemotaxis [5]. However, these cytokines could not be under divided into one of the canonical subclasses, as they did not contain the specific N-terminal cysteine residue. Therefore, these cytokines were classified into a newly defined fifth subclass, called the “chemokine-like function” (CLF) chemokines [5]. Most receptors for chemokines, called chemokine receptors, are G protein-coupled receptors. Therefore, upon chemokine binding, these receptors will activate G proteins and thus downstream intracellular signaling. However, also G protein-independent, atypical chemokine receptors have been described. These receptors mainly function as scavenger receptors for chemokines [6]. Chemokines and their receptors are widely expressed and are prominently present on cells that play a crucial role in atherosclerosis development like endothelial cells (ECs), smooth muscle cells (SMCs), and leukocytes [7]. Chemokines play an important role in all stages of atherosclerosis development (Fig. 1).

## Chemokines in atherosclerosis initiation

Atherogenesis generally starts with EC damage, resulting in an increased permeability of the endothelial layer, leading to the accumulation of lipids, especially low density lipoproteins (LDL) in the intima [8]. LDL in the subendothelial layer is very susceptible to oxidation, resulting in oxidized-LDL particles (oxLDL) [9]. This modified LDL will be taken up by resident macrophages and hydrolyzed into free cholesterol and fatty acids [10, 11]. Subsequently, free cholesterol will undergo re-esterification, forming cholesteryl esters [12]. Macrophages are not able to excrete these esters resulting in continuous intracellular accumulation, transforming macrophages into foam cells, which are characteristic of initial atherosclerosis. Besides macrophage activation, ox-LDL has also been shown to activate endothelial cells. A component of LDL, lysophosphatidic acid, has been shown to release CXCL1 from ECs [13], which has been shown to be important for the mobilization of monocytes and neutrophils to the site of inflammation via its receptor CXCR2 [13–15].

In response to the vascular inflammation caused by EC and macrophage activation, mainly monocytes will be attracted not only to the site of injury but also to other immune cells like neutrophils, T- and B-lymphocytes [9]. Monocytes in the circulation are present in two main subtypes, the classical (Ly6C^high^) and the nonclassical (Ly6C^low^) monocytes [16]. Classical monocytes are the main subtype that will migrate toward atherosclerotic lesions [17]. After this recruitment, monocytes will adhere to the vessel wall and transmigrate into the intima of the vessel by a process consisting of various interactions between adhesion molecules and chemokines [18]. These interactions are very complex, and although a lot of research has already been performed in this area of interest, dogmas continue to change, and there is still a lively debate about the precise role of the various chemokines in this process. This dynamics is clearly visible when we look at the literature from the last 10 years focusing on the recruitment of monocytes to atherosclerotic lesions. In 2007, Tacke et al. showed that CCR2, CCR5 and CX_3_CR1 are required for monocyte recruitment [19]. For this, they transferred atherosclerotic aortic arches from ApoE^−/−^ mice into the specific chemokine receptor knockout mice. More recently, however, using adoptive transfer experiments combined with pharmacological inhibition, Soehnlein et al. showed that not CCR2 or CX_3_CR1 but CCR1 and CCR5 are necessary for monocyte recruitment [15]. Surprisingly, looking at the role of CCR1 and CCR5 in general atherosclerosis development, their role appears to be contradicting. Mice deficient for CCR5 show reduced atherosclerosis development upon high fat diet feeding, although CCR1-deficient mice show clearly increased plaque development [20, 21]. Though these two receptors share ligands like CCL3 and CCL5, they can also both bind specific ligands, which might explain the differential outcome on atherosclerosis development. Another possible explanation for this differential involvement of CCR1 and CCR5 in atherosclerosis is the contrary effects on the Th1/Th2 balance, with CCR1 deletion favoring a proatherogenic Th1 response [22]. Although the adoptive transfer study did not show a role for CCR2 in monocyte recruitment, CCR2-deficient mice do show significantly reduced atherosclerosis development. This could however be explained by a reduction of monocyte release from the bone marrow in CCR2-deficient mice, rather than inflammatory recruitment effects [23, 24]. These data clearly indicate that chemokines do play an important role in monocyte recruitment, although the precise interactions still deserve further investigation (Fig. 1).

Recruited monocytes will start to adhere to the vessel wall in a so-called capture and rolling process. During this process, various chemokines, but especially also selectins present on the activated endothelium, play an important role [25]. Subsequently, monocytes will firmly adhere to the endothelium via interactions of endothelial vascular cell adhesion molecule 1 (VCAM1) with monocyte very late antigen 4 (VLA4) and endothelial intercellular adhesion molecule 1 (ICAM1) with monocyte lymphocyte function-associated antigen 1 (LFA1) [26]. Finally, monocytes will transmigrate across the endothelial layer again under the influence of chemokines [27]. Besides the influence of chemokines, also the permeability of the endothelial layer is very important in this phase. Therefore, junctional molecules like VE-Cadherin and junctional adhesion molecules (JAMs) are important in mediating this transmigration into the intima of the vessel wall [28]. In the intima, monocytes will differentiate into macrophages, mediated by macrophage colony-stimulating factor (M-CSF) [29]. These newly formed macrophages will again be exposed to oxLDL present in the intima which will as described before result in the formation of foam cells. This vicious circle of lipid accumulation and leukocyte recruitment will continuously stimulate atherosclerosis development.

## Chemokines in lesion progression and regression

Chemokines play a role not only in leukocyte recruitment and adhesion but also during plaque progression. As a result of the continuous accumulation of lipids and debris, lesional macrophages will eventually become apoptotic. Neighboring macrophages can take up the formed apoptotic debris in a process called efferocytosis [30]. However, as more and more debris will form upon lesion progression, neighboring macrophages will become stressed, impairing their efferocytotic capacity [31]. Apoptotic macrophages will then go into secondary necrosis, forming a necrotic core [30, 32]. It has been shown that CX_3_CR1 plays an important role in cell survival, as CX_3_CR1-deficient mice showed an increased apoptosis of lesional macrophages [33]. This effect on cell survival, rather than leukocyte recruitment was the underlying cause of the observed reduction in atherosclerosis development in CX_3_CR1-deficient mice [33].

Upon lesion progression, a fibrous cap is formed to protect the lumen from the necrotic core, which contains thrombotic factors. This cap mainly consist of collagen and elastin, produced by SMCs [34]. Fibrous cap thickness is often used as a measure of lesion stability, where lesions with a thicker cap are more stable and thus less prone to rupture. It has been shown that CXCL10 plays an important, although detrimental, role in this plaque stability [35]. Inhibition of CXCL10 results in relatively more SMCs, suggesting that CXCL10 itself will result in a thinner fibrous cap. This fibrous cap thinning can also be caused by matrix metalloproteases (MMPs), produced by lesional macrophages or SMCs, that can degrade the formed extracellular matrix [36]. The balance between production and degradation determines the thickness of the cap and thus stability of the lesion. Vulnerable lesions, consisting of a thin fibrous cap, are very likely to rupture and cause an occlusion of the blood vessel resulting in a cardiovascular event [32].

Platelets play a crucial role in this thrombus formation after the rupture of atherosclerotic lesions. Various chemokines are involved in the activation and aggregation of platelets [37]. CXCL12 is one of the chemokines able to induce platelet aggregation [38]. Blocking studies additionally showed that also the receptor for CXCL12 and CXCR4, expressed on platelets, is crucially involved in the platelet aggregation effects of CXCL12 [38]. Additionally, it has been shown that CXCL12 is capable of stimulating platelet migration and transmigration [39]. The macrophage-derived chemokines CCL17 and CCL22 have also been demonstrated to induce platelet activation via their receptor CCR4 [37].

Chemokines also play an important role in the regression of atherosclerotic lesions. Various studies have already described that CCR7 is crucial for the egress of macrophages, resulting in plaque regression [40–42].

Together, these results show a clear involvement of the chemokine system in all stages of atherosclerosis development (Fig. 1). However, further research is still needed to elucidate various contradicting findings and to fully understand the precise role of these complex interactions. Recent years, CXCL12 and macrophage migration inhibitory factor (MIF) have gained a lot of interest in the field of atherosclerosis research. Therefore, the remainder of this review will discuss the current knowledge about these chemokines and their receptors in relation to atherosclerosis and the clinical implications.

## CXCL12—general signaling and function

CXCL12 or stromal cell-derived factor 1 (SDF-1) is a member of the CXC chemokine family [43]. This chemokine consists of various isoforms. The two classical isoforms, which are expressed throughout the body and are functionally indistinguishable, are CXCL12-α and CXCL12-β [44]. However, also CXCL12-γ, CXCL12-δ, CXCL12-ε, and CXCL12-φ isoforms exist that are not as widely expressed and until now much less studied [44]. CXCL12-deficient mice are embryonically lethal, indicating the general physiological importance of this chemokine [45]. CXCL12 especially plays an important role in stem- and progenitor cell mobilization as high levels of CXCL12 will retain cells in the bone marrow [46]. In the clinic, this is already being exploited by modulating CXCL12 by using granulocyte colony-stimulating factor (G-CSF) to induce stem cell recruitment, reviewed in [47].

**Fig. 2:**
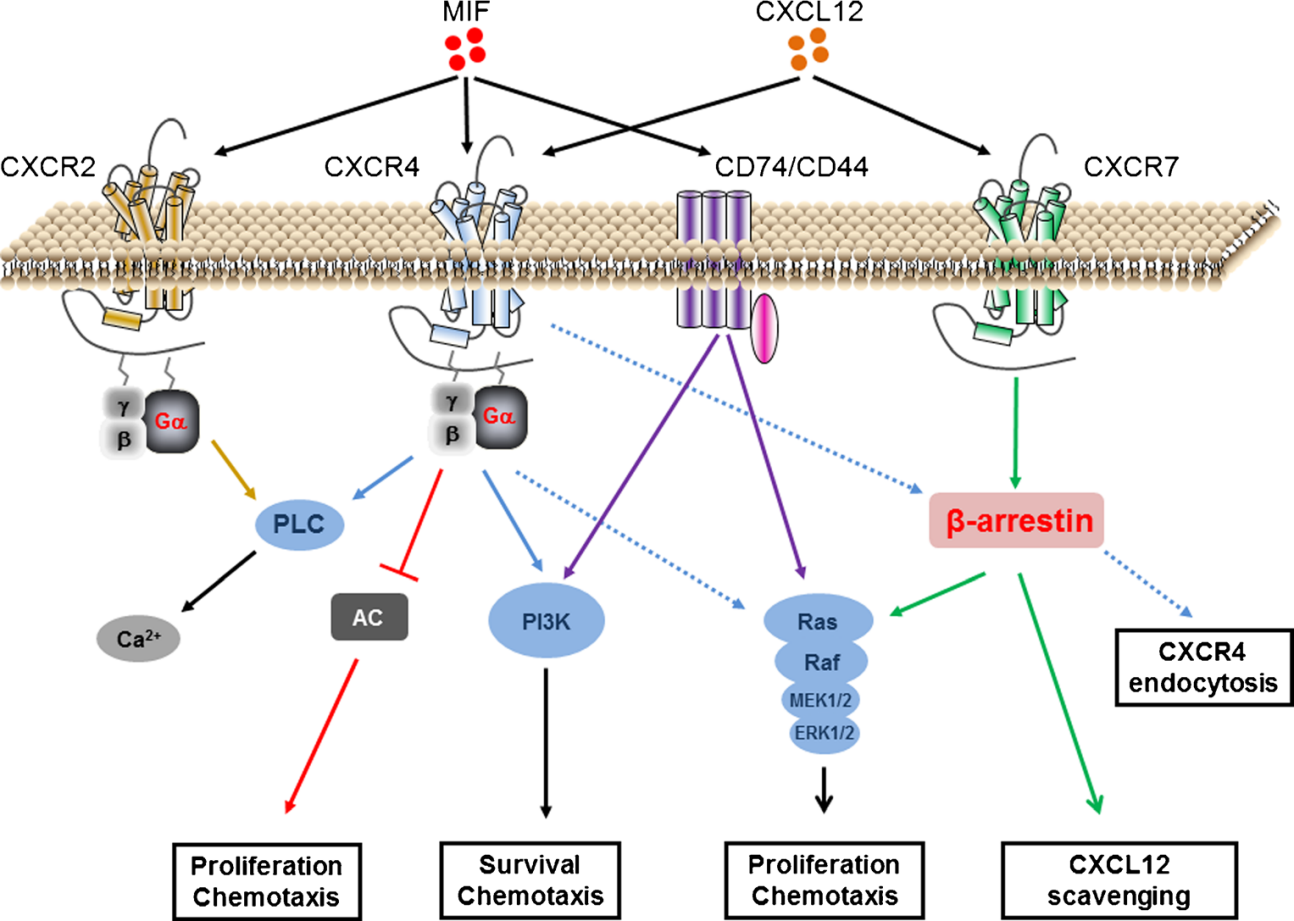
Signaling by MIF and CXCL12. MIF binds to CXCR2, CXCR4, and CD74 receptors, whereas CXCL12 binds to CXCR4 and CXCR7. CXCR2 and CXCR4 induce G protein-coupled signaling leading to effects on survival, proliferation, and chemotaxis. CD74/CD44 mediates similar effects in a G protein-independent manner. β-Arrestin-mediated signaling induced by CXCR4 will result in receptor endocytosis. CXCR7 is not a G protein-coupled receptor but can induce β-arrestin signaling resulting in CXCL12 scavenging. *AC* adenylyl cyclase, *MIF* macrophage migration inhibitory factor, *PI3K* phosphatidylinositide 3-kinase, *PLC* phospholipase C

CXCR4 is expressed in a wide variety of cells and is the main receptor for CXCL12 [48, 49]. Since CXCR4 is a G protein-coupled receptor, it can mediate intracellular signaling via G proteins [48]. G proteins consist of various subunits. Signaling via CXCR4 seems to be mainly mediated by the Gα_i_ subunit. Upon binding of CXCL12 to this receptor, G proteins not only dissociate and can trigger MAPK and PI3K signaling but also inhibit adenylyl cyclase [50]. Other G protein subunits, the Gβγ dimer, can also be activated upon ligand binding, resulting in the mobilization of intracellular calcium by phospholipase C activation [50]. Finally, CXCR4 has also been shown to induce β-arrestin recruitment [51]. Recruitment of β-arrestin to CXCR4 will mediate receptor desensitization by the endocytosis of the receptor (Fig. 2).

Another receptor for CXCL12, with even a 10-fold higher affinity compared with CXCR4, is CXCR7 [52]. However, CXCR7 does not induce intracellular signaling using G proteins like CXCR4 upon binding of CXCL12. The main effect of CXCR7 activation is β-arrestin recruitment, resulting in the internalization and subsequent degradation of the receptor with its bound ligand [53, 54]. In this way, CXCR7, acting as a decoy receptor, controls the availability of extracellular CXCL12 and has been implicated in cell growth and survival [55] (Fig. 2). It has been shown that both receptors for CXCL12 also interact with each other in an antagonistic manner [56, 57].

## CXCL12 in atherosclerosis

In recent years, more research has focused on determining the role of CXCL12 in atherosclerosis development. Mice that were injected with CXCL12 developed more stable atherosclerotic lesions, characterized by a thicker fibrous cap [58]. This beneficial effect on atherosclerosis could be explained by the increased recruitment of SMC progenitor cells (SPCs) to these lesions. Beneficial effects of the CXCL12/CXCR4 axis were also observed using endothelial progenitor cells (EPCs) [59], indicating that the effects on progenitor mobilization of this axis work atheroprotective.

CXCL12 and its main receptor CXCR4 are expressed on various atherosclerosis-related cell types, like ECs, SMCs, and leukocytes [60–63]. The exact causal role of cell-type-specific CXCL12/CXCR4 expression remains to be elucidated. However, already various studies have showed clear associations of cell-type-specific expression of CXCL12 with atherosclerosis development. In macrophages, for example, the CXCL12/CXCR4 axis has been implicated in macropinocytosis and could thus play an important role in the accumulation of oxLDL and foam cell formation [64]. It has also been implicated in neutrophil release from the bone marrow as well as in the clearance of senescent neutrophils from the circulation [65, 66]. Treating atherosclerotic prone mice with a CXCR4 inhibitor, AMD3100, resulted in increased atherosclerotic lesion areas due to increased neutrophil mobilization [67]. Furthermore, oxLDL stimulation of ECs resulted in an increased release of CXCL12 [68], and chemotaxis of lymphocytes was stimulated by the CXCL12/CXCR4 axis [69–72]. These results clearly show that the CXCL12/CXCR4 axis influences many cell types and plays an important role in atherogenesis. The alternative receptor for CXCL12 and CXCR7 has also been implicated in atherosclerosis development, as activation of this receptor reduced lesion formation, by increasing the VLDL uptake in adipose tissue [73].

In humans, there are also already clear associations between CXCL12 and atherogenesis revealed by genome-wide association studies [43] and immunohistochemical stainings of human lesions, showing more CXCL12 expression in atherosclerotic lesions, compared to normal vessels [38]. This role for CXCL12 in atherogenesis is also confirmed in other human studies, although the clinical findings remain rather contradictory. Angina patients show decreased plasma levels of CXCL12, compared to healthy controls, with a decreased surface expression of CXCR4 in peripheral blood mononuclear cells, indicating atheroprotective effects of CXCL12 [74]. In contrast, angina patients show increased platelet expression of CXCL12, compared to healthy controls, and plasma CXCL12 levels correlate with platelet activation, suggesting more pro-atherogenic effects of CXCL12 [75]. Future research is necessary to further elucidate these contradicting clinical findings. As platelet-derived CXCL12 expression occurs within 30 min after vessel injury, this chemokine might be a suitable candidate to function as early biomarker [76, 77].

## MIF—general signaling and function

The MIF chemokine is missing the characteristic N-terminal cysteines and therefore part of the CLF chemokine family [78]. Originally, T cells were identified as the main source of MIF, although in recent years, various studies have shown MIF expression in many other leukocytes, like monocytes, neutrophils, dendritic cells, and B cells [79]. MIF has shown to be an pro-inflammatory chemokine, as it not only induces the production of various cytokines and nitric oxide but also overrides the immunosuppressive effects of glucocorticoids [80].

CD74, a MHC class II chaperone, was the first identified receptor for MIF [81]. Although MIF binds with high affinity to this receptor, it is not able to induce intracellular signaling. For this, co-receptors are necessary like CD44, CXCR2, or CXCR4 [5]. The CD74/CD44 complex has been linked to cell proliferation and survival via MAPK and PI3K signaling, respectively [82, 83]. Additionally, CD74 has been shown to play a role on monocyte and neutrophil chemotaxis and arrest [84]. As the CD74/CXCR2 complex is implicated in MIF-mediated chemotaxis, these receptors are associated with atherosclerosis [85]. CXCR4 by itself can also act as receptor for MIF and seems to be especially important for MIF mediated T cell recruitment [84]. In contrast, CXCR2 is the MIF receptor responsible for not only monocyte recruitment but also integrin activation, a critical step in firm adhesion of monocytes to the endothelium [84]. CXCR2 is also a G protein-coupled receptor and is capable of inducing intracellular signaling [78] (Fig. 2).

## MIF in atherosclerosis

It has been shown that MIF expression in atherosclerosis-related cells, like ECs, SMCs, monocytes, and T cells, positively correlates with atherosclerosis progression, implicating MIF in atherogenesis [86–88]. MIF has also been shown to increase the adhesion of monocytes to aortic ECs in in vitro adhesion assays under flow [89]. MIF also interfered with the expression of adhesion molecules and cytokines that are important mediators of leukocyte recruitment, as RNA-silencing of MIF decreased the expression of E-selectin, ICAM1, VCAM1, IL-8, and CCL2 [90]. Even more convincing results are given by Bernhagen et al., where they show that MIF triggers monocyte, neutrophil, and T cell arrest and chemotaxis in an integrin-dependent manner [84]. The role of MIF in atherogensis was also confirmed in mouse models, where MIF deficiency resulted in a reduction of lipid deposition and atherosclerotic lesion size compared to wild-type mice [91]. MIF inhibition by neutralizing antibodies showed similar effects on atherogenesis, mainly mediated by a reduction in lesional inflammation [86]. MIF is also associated with plaque destabilization, as it has been shown that MIF stimulates not only oxLDL uptake by macrophages but also MMP secretion [91]. The receptor for MIF, CXCR2, has also been implicated in atherosclerosis as CXCR2 deficiency results in reduced lesion size and lesional macrophage content [92]. Together, these results clearly show pro-atherogenic effects of MIF and its receptors.

MIF is also abundantly expressed in every stage of plaque development in humans [87]. However, MIF seemed to play a more important role in vulnerable lesions as it induces MMP-1 expression and activity in SMCs, leading to fibrous cap thinning [93]. Plasma levels of CVD patients also showed increased MIF levels, compared to healthy controls, associating MIF with CVD development in humans [94]. In CVD patients with impaired glucose tolerance or type 2 diabetes mellitus, high plasma MIF levels were even shown to be an independent risk factor for future cardiovascular events [95]. This pro-atherogenic role of MIF has also been confirmed by human epidemiological studies, showing that a single nucleotide polymorphism in the MIF gene associated with a significantly increased risk for MI [96]. In support of this, MI patients have increased plasma MIF levels as early as 4 to 6 h after acute MI, making MIF a suitable candidate to be used as early detection marker of MI [97]. However, a prospective study has warranted caution to use MIF as a biomarker as there was only a very weak association of MIF with risk of MI or death due to CVD in humans without prior history of CVD [98].

## Concluding remarks

It is well known that chemokines play an important role in atherosclerosis and thus CVD. However, the interactions not only between the various chemokines but also between the various involved cell types in atherogenesis remain very complex. As seen over the last years, dogmas with regard to this interaction are still highly dynamic, and further research will be necessary to fully elucidate these interactions. There are already some potential therapies developed, targeting the chemokine system. However, the development of effective but safe therapeutics has shown to be rather challenging [99]. For MIF, also several inhibitors have already been developed showing beneficial effects in various inflammatory models [100]. Future research should further determine the therapeutic potential of chemokines in atherosclerosis. An important part of this will be the identification of cell-type-specific effects of chemokines and their receptors, creating opportunities for more specific therapeutic targets.
